# Grape Seed Proanthocyanidin Extract–Mediated Regulation of STAT3 Proteins Contributes to Treg Differentiation and Attenuates Inflammation in a Murine Model of Obesity-Associated Arthritis

**DOI:** 10.1371/journal.pone.0078843

**Published:** 2013-11-05

**Authors:** Joo Yeon Jhun, Su-Jin Moon, Bo Young Yoon, Jae Kyung Byun, Eun Kyung Kim, Eun Ji Yang, Ji Hyeon Ju, Yeon Sik Hong, Jun Ki Min, Sung Hwan Park, Ho Youn Kim, Mi-La Cho

**Affiliations:** 1 Conversant Research Consortium in Immunologic disease, College of Medicine, The Catholic University of Korea, Seoul, South Korea; 2 Rheumatism Research Center, College of Medicine, The Catholic University of Korea, Seoul, South Korea; 3 Division of Rheumatology, Department of Internal Medicine, College of Medicine, The Catholic University of Korea, Seoul, South Korea; 4 Department of Internal Medicine, Inje University, Ilsan Paik Hospital, Goyang, South Korea; New York University, United States of America

## Abstract

Grape seed proanthocyanidin extract (GSPE) is a natural flavonoid that exerts anti-inflammatory properties. Obesity is an inflammatory condition and inflammatory cells and their secretion of pro-inflammatory molecules contribute to the pathogenesis of obesity. Rheumatoid arthritis (RA) is a chronic autoimmune disease that is characterized by inflammation of joints lined by synovium. Previously, we demonstrated that obesity augmented arthritis severity in collagen induced arthritis (CIA), a murine model of human RA. Here, we investigated whether oral administration of GSPE showed antiobesity and anti-arthritic effects in high-fat diet-induced obese (DIO) mice and in obese CIA mice, respectively. The pathophysiologic mechanisms by which GSPE attenuates weight gain and arthritis severity *in vivo* were also investigated. In DIO mice, GSPE administration significantly inhibited weight gain, reduced fat infiltration in liver and improved serum lipid profiles. The antiobesity effect of GSPE was associated with increased populations of regulatory T (Treg) cells and those of decreased Th17 cells. Decrease of Th17 cells was associated with significant inhibition of their key transcriptional factors, pSTAT3^Tyr705^ and pSTAT3^Ser727^. On the contrary, GSPE-induced Treg induction was associated with enhanced pSTAT5 expression. To identify the anti-arthritis effects of GSPE, GSPE was given orally for 7 weeks after type II collagen immunization. GSPE treatment significantly attenuated the development of autoimmune arthritis in obese CIA model. In line with DIO mice, GSPE administration decreased Th17 cells and reciprocally increased Treg cells by regulating STAT proteins in autoimmune arthritis model. The expressions of pro-inflammatory cytokines and nitrotyrosine in synovium were significantly inhibited by GSPE treatment. Taken together, GSPE functions as a reciprocal regulator of T cell differentiation – suppression of Th17 cells and induction of Tregs in both DIO and obese CIA mice. GSPE may act as a therapeutic agent to treat immunologic diseases related with enhanced STAT3 activity such as metabolic disorders and autoimmune diseases.

## Introduction

Grape seed proanthocyanidin extract (GSPE), a family of natural polyphenolic flavonoids, is present in a wide variety of plant foods such as fruits, berries, beans, nuts, cocoa, and wine [1]. These naturally occurring antioxidants have been shown to exert a broad spectrum of biological, pharmacological and therapeutic activities against free radicals and oxidative stress [2]. Epidemiological studies have strongly suggested that regular consumption of proanthocyanidins may prevent the risk of cardiovascular diseases [3]. In addition to its antioxidants properties, GSPE has been described as an anti-inflammatory agent [4], and shown to increase mitochondrial biogenesis [5]. In fact, GSPE provided significantly better scavenging activity toward biochemically generated superoxide anion, when compared to vitamins C and E [6]. Furthermore, recent studies in animals have demonstrated potent anti-inflammatory properties of proanthocyanidins on experimental inflammation [7].

Obesity is a metabolic disease that is characterized by low-grade chronic inflammation. Population studies have shown a strong correlation between the level of pro-inflammatory biomarkers such as C-reactive protein, interleukin-6 (IL-6), and tumor necrosis factor (TNF)-α, and perturbations in glucose homeostasis, obesity and atherosclerosis [8], suggesting the possibility of a common pathophysiologic link between autoimmunity and obesity. Mediators linking adipose tissue, inflammation, and immunity are adipocytokines such as adioponectin, leptin, resistin, and visfatin [9]–[11]. Infiltrating macrophages of the adipose tissue may be sources of these adipocytokines [8]. Leptin is the most pivotal adipocytokine that induces inflammation by increasing TNF-α, IL-6, IL-12 [12], and reactive oxygen species (ROS) [13] and by immune deviation toward Th1 differentiation [14]. Recent studies have reported that T cells are regulated in adipose tissues ant may contribute to obesity-induced inflammation [15]. Interestingly, emerging studies have indicated that obesity selectively promotes the expansion of IL-17 producing CD4^+^ T (Th17) cells in adipose tissues, exacerbating autoimmunity in murine models [16]. These results may indicate obesity having not only metabolic problems such as insulin resistance and hyperlipidemia, but also immunologic problems. Thus, it could be postulated that anti-inflammatory agents exhibit a therapeutic property on the development of obesity – in aspect of metabolic disorder.

In our previous studies, GSPE treatment exhibited chondroprotective and antinociceptive properties in a rat model of osteoarthritis through antioxidative effect [17] and also showed anti-inflammatory effects in mice with collagen-induced arthritis (CIA), a murine model of rheumatoid arthritis (RA) [18]. These results suggest that GSPE has therapeutic effects against metabolic diseases and autoimmune diseases. In line with previous studies, we also recently found the possible relationship between metabolic disorders and inflammatory diseases, where obesity aggravated joint inflammation in CIA mice [19]. In that study, obesity plays an additive role in the development of inflammation through type II collagen specific T cell differentiation – Th17 cells differentiation and IL-17 is a pivotal cytokine that accelerates joint inflammation in obese CIA mice. Thus, we hypothesized that anti-inflammatory agents could treat obesity through an immunologic mechanism in the aspect of Th17 cells differentiation. If so, GSPE could be a therapeutic agent not only in obesity itself, but also in obese CIA mice, our new murine model of autoimmune arthritis [19]. Here, we investigated whether oral administration of GSPE exerts the antiobesity and antiarthritic properties in high-fat-diet-induced obese (DIO) mice and obese CIA mice. We also investigated the underlying pathophysiologic mechanisms by which GSPE treatment attenuates weight gain and arthritis severity in mice.

## Materials and Methods

### Animal

C57BL/6 mice(SLC, Inc., Shozuoka, Japan), 4 weeks old, were housed in polycarbonate cage and fed 60 Kcal fat-derived calories or standard mouse chow (Ralston Purina, St Louis, MO, USA) and water ad libitum. All experimental procedures were examined and approved by the Animal Research Ethics Committee of the Catholic University of Korea.

### Induction of obese CIA and GSPE treatment

C57BL/6 mice (4weeks) were purchased from Charles River Breeding Laboratories. CIA was induced as previously described [20]. The CII immunization was done the first time when the mouse weighed 30 g. In brief, complete Freund's adjuvant (CFA; Chondrex, Redmond, WA) was prepared by grinding 2 mg or 10 mg of heat-killed M. tuberculosis (H37Ra; Difco Laboratories, Detroit, MI) in 2 ml of incomplete Freund's adjuvant (IFA; Chondrex). An emulsion was formed by dissolving 2 mg/ml chick collagen type II (CII; Chondrex, Redmond, WA) overnight at 4°C in 0.5 M acetic acid, followed by mixing this with an equal volume of CFA. The mice were intradermally injected with the emulsion at two sites at the base of the tail and a slightly more anterior location. A second injection as a booster was done 14 days after the primary immunization. GSPE was kindly provided by Hanlim Pharmaceutical Company (Seoul, Korea). GSPE, dissolved in saline, was given orally three times per week for 7 weeks after the first immunization. The GSPE dose was 300 mg/kg.

### Histological assessment of arthritis

The mouse joint tissues were fixed in 4% paraformaldehyde, decalcified in EDTA bone decalcifier, and embedded in paraffin. Seven-micrometer sections were prepared and stained with H&E, Safranin O, and toluidine blue to detect proteoglycans. The sections were dewaxed using xylene; then they were dehydrated in a graded series of alcohols. The endogenous peroxidase activity was quenched with methanol and 3% H_2_O_2_. Immunohistochemostry was performed using the Vectastain ABC kit (Vector Laboratories, Burlingame, CA). The tissue were first incubated with the primary anti-IL-17, anti-IL-1, anti-TNF-α, anti-Nitrotyrosine (all for Santa Cruze Biotechnology, Santa Cruz, CA); anti-IL-6 (Abcam), mouse IgG isotype (for nitrotyrosine), or goat isotype (for TNF-α) or rabbit IgG isotype (for IL-1, L-6 and IL-17; all from Santa Cruz Biotechnology) overnight at 4°C; and abiotinylated secondary linking Ab and a streptavidin-peroxidase complex for 1 h. The final color product was developed using 3,3-diaminobenzidine chromogen (DAKO, Carpinteria, CA). The sections were counterstained with hematoxylin. Images were captured using a DP71 digital camera (Olympus, Center Valley, PA) attached to an Olympus BX41 microscope at ×200 magnification. For histologic evaluation of CIA, sections were evaluated in a blind manner, as has been described previously. The scores were evaluated as previously described [21].

### Confocal microscopy

For confocal staining, 7 µm tissue sections of spleens were stained using FITC conjugated anti-CD4, PE-conjugated anti-IL-17, PE-conjugated anti-pSTAT3^Ser727^, PE-conjugated anti-p-STAT3(Tyr705), PE-conjugated anti-p-STAT5, PE conjugated anti-CD4, APC-conjugated anti-CD25, and FITC conjugated anti-Foxp3 (all from eBiosciences, San Diego, CA, USA). Stained sections were analyzed using a Zeiss microscope (LSM 510 Meta; Carl Zeiss, Oberkochen, Germany) at 400 magnification.

### Clinical assessment of arthritis

The severity of arthritis was determined by three independent observers. The mice were observed three times a week for the onset and severity of joint inflammation for up to 8 weeks after the primary immunization. The severity of arthritis was assessed on a scale of 0–4 with the following criteria, as described previously [22]: 0 =  no edema or swelling, 1 =  slight edema and erythema limited to the foot or ankle, 2 =  slight edema and erythema from the ankle to the tarsal bone, 3 =  moderate edema and erythema from the ankle to the tarsal bone, and 4 =  edema and erythema from the ankle to the entire leg. The arthritic score for each mouse was expressed as the sum of the scores of three limbs. The hind paw into which type II collagen + incomplete Freund's adjuvant was injected was excluded. The highest possible arthritis score for a mouse was thus 12. The mean arthritis index was used to compare the data among the control and experimental groups.

### Measurement of type II collagen–specific antibodies

The serum levels of type-II-collagen-specific Total IgG and IgG2a were measured by enzyme-linked immunosorbent assay (ELISA), as previously described [22], with minor modifications. Briefly, micro-titer plates were coated with type II collagen (4 µg/mL in PBS) at 4 °C overnight, followed by a blocking step for 30 min at room temperature. Serum samples were then diluted 1∶100 in Tris-buffered saline (pH 8.0) containing 1% bovine serum albumin and 0.5% Tween-20, and incubated in the micro-titer plates for 1 h, after which the plates were washed five times. The concentrations of Total IgG and IgG1 were measured using mouse Total IgG and IgG2a ELISA Quantitation Kits (Bethyl Laboratories, Montgomery, TX), respectively. Standard serum from arthritic mice was added to each plate in serial dilutions, and a standard curve was constructed to assign arbitrary units to the levels anti-type-II-collagen Total IgG and IgG2a. The absorbance values were determined with an ELISA microplate reader operating at 450 nm.

### T cell Proliferation

The mouse spleens were collected for cell preparation and they were washed twice with PBS. The spleens were minced and the red blood cells were lysed with 0.83% ammonium chloride. The cells were filtered through a cell strainer and then they were centrifuged at 1300 rpm at 4°C for 5 min. The cell pellets were resuspended in RPMI 1640 medium. Splenocytes of obese-CIA and GSPE-treated mice were cultured at 2×10^5^ cells/well in 96-well flat bottom plates were cultured in the absence or presence of plate-bound CD3 0.5 µg/ml, 1 µg/ml and 2 µg/ml at 37°C for 72 h. During the last 16–18 h of the 3 d assay, cells were pulsed with 1 µCi of [3H]-thymidine (GE Healthcare, Little Chalfont, UK) per well. The incorporation of [3H]-thymidine was determined using a Betaplate scintillation counter (Perkin-Elmer, Wellesley, MA).

### Biochemical analyses

Blood samples were collected from all treated and control mice 7 weeks after the immunization and stored at −70 °C until use. The levels of total serum Cholesterol were measured using commercial kits Wako Co. (Osaka Japan), and Glucose, LDL-Cholesterol s were measured using commercial kits from Asan Pharmaceutical Co. (Hwangseong-gi Gyeonggi-do, Korea)

### Intracellular staining and flow cytometry

A population of Foxp3^+^Treg cells and Th17 cells were observed in splenocytes of DIO mice with or without GSPE treatment. For intracellular staining, cells were stimulated with 25 ng/ml PMA (Sigma-Aldrich, St Louis, MO) and 250 ng/ml ionomycin (Sigma-Aldrich) and Golgi Stop (BD Biosciences, San Diego, CA) were added for 4 hr. Cells were stained with Percp-conjugated anti-CD4 Ab (BD Pharmingen) then stained with FITC-conjugated anti-IL-17 (all from eBiosciences), followed by fixation and permeabilization using the Cytofix/Cytiperm Plus Kit (BD Biosciences) according to the manufacturer's instructions. For analysis of Treg cells, Splenocyte were surface labeled with CD4 and CD25, followed by fixation, permeabilization and intracellular staining with Foxp3. Treg-cell staining was performed using the eBioscience Foxp3 staining kit (eBioscience, San Diego, CA). Cells were stained with PerCP-conjugated anti-CD4 Ab (BD Pharmingen) then stained with APC-conjugated anti-CD25 and PE-conjugated anti-Foxp3(all from eBiosciences), followed by fixation and permeabilization using the Mouse Foxp3 Buffer Set (BD Biosciences) according to the manufacturer's instructions. All samples operated on a FACSCalibur (BD Pharmingen), where the data was analyzed using the FlowJo software (Tree Star, Ashland, OR,USA).

### Analysis of gene expression by real-time quantitative PCR

Total RNA was extracted using TRIzol (Molecular Research Center, Cincinnati, OH, USA). 2 ug of total RNA were reverse transcribed using the Superscript Reverse Transcription system (Takara, Shiga, Japan). Quantitative real-time PCR (qRT-PCR) was performed with LightCycler FastStart DNAmaster SYBR green I (Takara) fluorescent dye using an ABI PCR machine. Primers for IL-17(forward: 5′- CCT CAA AGC TCA GCG TGT CC-3′, reverse: 5′-GAG CTC ACT TTT GCG CCA AG-3′), forkhead box P3 (Foxp3; forward: 5′-GGC CCT TCT CCA GGA CAG A-3′, reverse: 5′-GCT GAT CAT GGC TGG GTT GT -3′), Retinoic acid-related orphan receptor (RORγt; forward: 5′-TGT CCT GGG CTA CCC TAC TG-3′, reverse: 5′-GTG CAG GAG TAG GCC ACA TT-3′, Runt-related transcription factor 1 (RUNX1; f orward: 5′-TAC CTG GGA TCC ATC ACC TC-3′, reverse: 5′-GAC GGC AGA GTA GGG AAC TG-3′), IL-21 (forward: 5′- AAG ATT CCT GAG GAT CCG AGA AG -3′, reverse: 5′- GCA TTC GTG AGC GTC TAT AGT GTC -3′), Suppressor of cytokine signaling-3 (SOCS3; forward: 5′- CTC AAG ACC TTC AGC TCC AA-3′, reverse: 5′- TTC TCA TAG GAG TCC AGG TA-3′) and β-actin (forward: 5′- GAAATCGTGCGTGACATCAAAG-3′, reverse: 5′- TGTAGTTTCATGGATGCCACAG-3′) were designed using Primer Express (Applied Biosystems, Foster City, CA).

### Statistical analysis

Differences between treatment groups were tested for statistical significance with Mann-Whitney *U*-test using GraphPad Prism 5 software. The results are expressed as means ± S.D. (or means ± S.E.M). The data were considered significantly different at *P*<0.05 (2-tailed).

## Results

### Antiobesity effect of GSPE in mice with high-fat diet-induced obesity (DIO)

**Figure 1:**
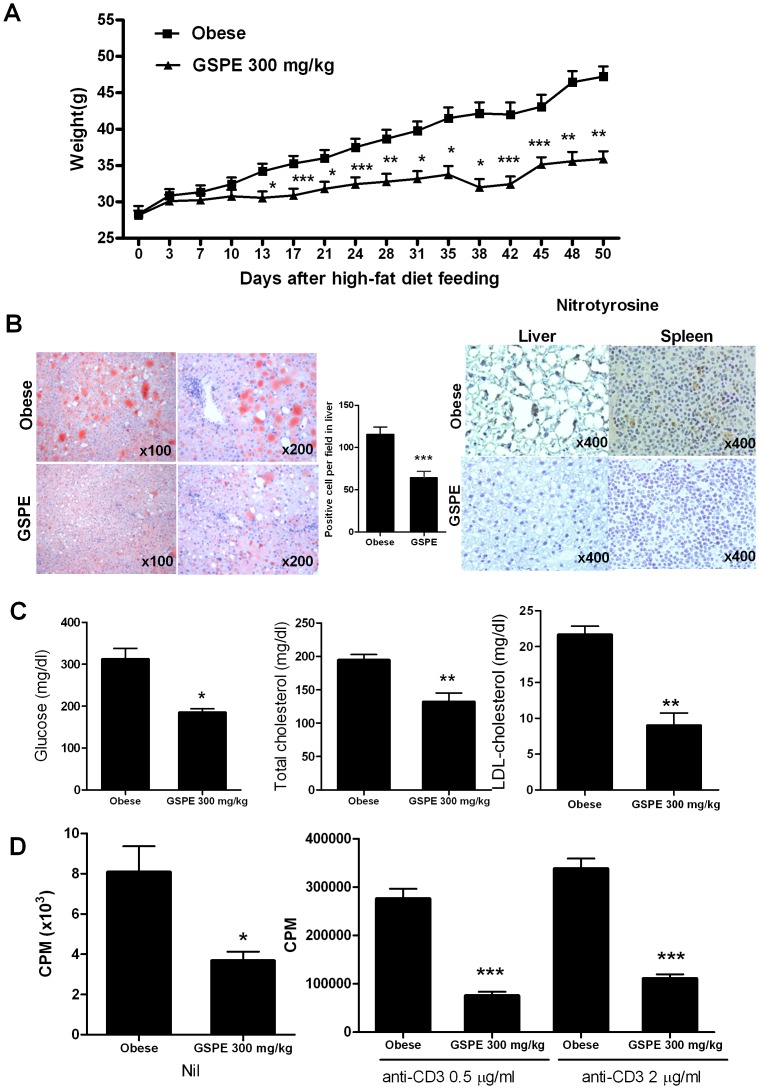
*In vivo* treatment with GSPE in mice significantly prevented obesity induced by a high-fat diet (HFD). The mice received continuous feeding of a HFD after 4 weeks of age and euthanized at 50 days (11 weeks of age) after feeding (n = 6 for each group). (A) Weight gain was significantly attenuated in mice administered with GSPE (300 mg/kg) during the experimental period when compared to control mice. (B) Representative photomicrographs (100×) of liver sections from control or GSPE-treated mice stained with Oil Red O. Red staining indicates lipid deposition (*left panel*). The graphs show the number of positive cells (*middle panel*). Representative immunohistochemical staining for nitrotyrosine in liver and spleen isolated from each group (original magnification, 200×) (*right panel*). (C) Glucose level and lipoprotein profiles were determined in sera that were isolated at 50 days after feeding. Values are the means ± SD. (D) Splenocytes of each group of mice were cultured with or without 0.5 µg/ml, or 2 µg/ml of anti-CD3 mAb for 72 hrs. The proliferative responses were determined by [^3^H] thymidine incorporation assay. The data presents the mean counts per min (cpm) ± SD. *P<0.05, ** P<0.01, *** P<0.001 indicated significant differences from the control.

To induced DIO model, C57BL/6 mice were fed a high fat diet (60 Kcal). When the mice weighed 25 g, they were given orally GSPE or control (saline). Since 13 days after feeding with high-fat diet, GSPE-treated DIO mice group had shown a significantly attenuated weight gain (**Figure 1A**). GSPE treatment significantly suppressed liver lipid content, demonstrated by Oil red O staining, when compared with control DIO mice (**Figure 1B** **, left panels**). We also investigated the expressions of nitrotyrosine, a oxidative stress marker, in liver and spleen tissues of each group of mice with DIO. Oral administration of GSPE in obese mice profoundly reduced the numbers of nitrotyrosine-expressing cells in liver as well as spleens, suggesting its antioxidative effects (**Figure 1B** **, right panels**). To assess the effects of GSPE on metabolic profiles, glucose, cholesterol and low-density lipoprotein (LDL) cholesterol levels were measured in mice. Of interest, plasma glucose, total cholesterol, and LDL-cholesterol levels were significantly decreased in GSPE-treated obese mice (**Figure 1C**).

### Altered T cell responses in DIO mice by GSPE administration

We investigated whether GSPE treatment affects immune responses regarding cellular proliferation rate. The results demonstrated that splenocytes that were isolated from GSPE-treated DIO mice showed lower proliferative capacity, compared with those of control mice (**Figure 1D, left panel**). To determine whether GSPE treatment affects T cell proliferative responses, splenocytes of each group of mice were stimulated with anti-CD3 antibody (0.5 µg/ml or 2 µg/ml). Then, the proliferative responses were estimated by thymidine incorporation assay. The cells obtained from spleens of DIO mice with GSPE treatment showed decreased T cell proliferation, as compared with those cells obtained from the control mice (**Figure 1D, right panel**).

### GSPE treatment induces Foxp3^+^ Treg differentiation and reciprocally represses Th17 differentiation in mice with obesity induced by high-fat diet

**Figure 2:**
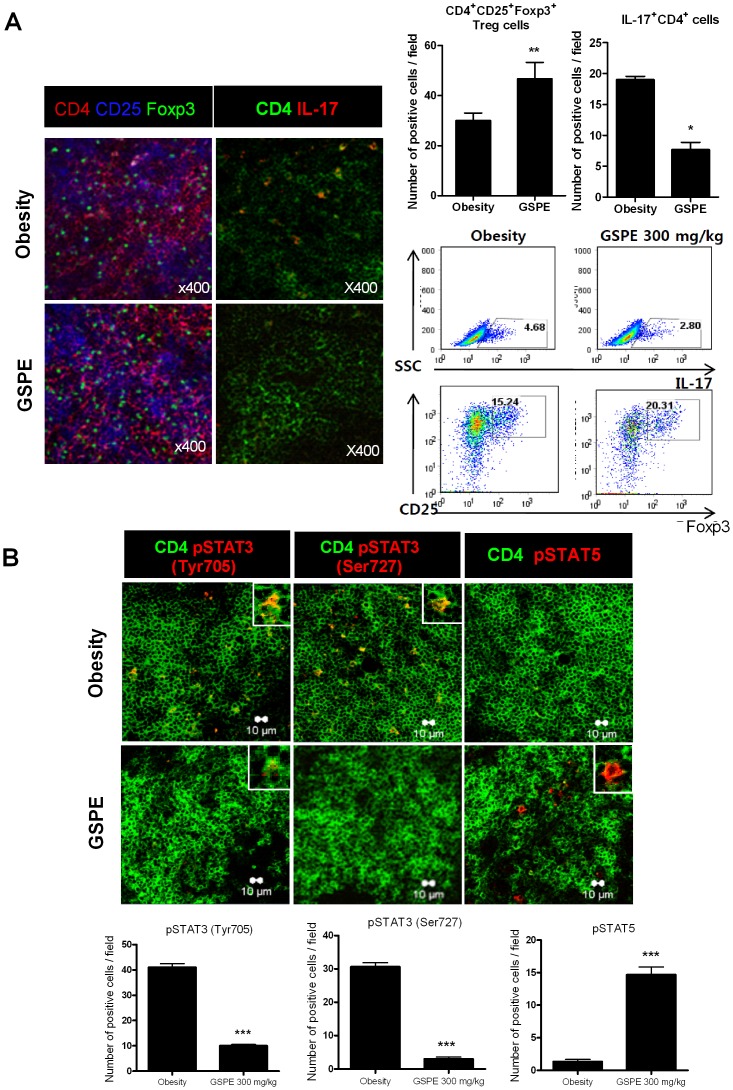
Reciprocal effects of GSPE on Th17 and Foxp3^+^ Treg cells population in obesity induced by a HFD. (A) Spleen tissues from each mouse were stained for CD4^+^CD25^+^Foxp3^+^ Treg cells and CD4^+^IL-17^+^ Th17 cells using monoclonal antibodies against CD4 (red), CD25 (blue), and Foxp3 (green) (*left image*) or CD4 (green) and IL-17 (red) (*right image*) (original magnification, 400×). Each confocal image is representative of five fields of view and three separate experiments. CD4^+^CD25^+^Foxp3^+^ T cells and CD4^+^IL-17^+^ T cells were enumerated visually at higher magnification (projected on a screen) by four individuals, and the mean values are presented in the form of a histogram (*right upper panel*). The populations of Th17 and Treg cells in spleens of each group of mice were determined by flow cytometry (*right lower panel*). *P<0.05, **P<0.01 *versus* the control group. (B) Spleens from mice in each group were examined by immunofluorescence staining with monoclonal antibodies against CD4 (green) and pSTAT3^Tyr705^ (red) (*left image*), or CD4 (green) and pSTAT3^Ser727^ (red) (*middle image*), or CD4 (green) and pSTAT5 (red) (*right image*). The cell populations were analyzed using laser confocal microscopy (original magnification, 400×). The graphs show the number of positive cells (*lower panel*). Values are the means ± SD. ***P<0.001 compared to the control mice.

We enumerated CD4^+^CD25^+^Foxp3^+^ Treg cells and CD4^+^IL-17^+^ Th17 cells in spleen tissues from DIO mice treated with or without GSPE. The results demonstrated that spleen tissues from obese mice treated with GSPE showed increases in the number of Foxp3^+^ Treg cells and reciprocally decreases in the number of Th17 cells, compared with control DIO mice (**Figure 2A**). The results of the flow cytometric analysis are consistent with those of confocal study. STAT3 is an essential transcription factor that is involved in Th17 differentiation, activation, proliferation and survival [23]. Hence, the expression levels of STAT3 and its phosphorylated forms in splenocytes were evaluated. The confocal microscopy results demonstrated that the expression of both STAT3 phosphorylated at tyrosine 705 (pSTAT3^Tyr705^) and STAT3 phosphorylated at serine 707 (pSTAT3^Ser705^) were decreased by treatment with GSPE (**Figure 2B**). On the opposite side of STAT3, STAT5 is the major transcription factor for the differentiation of Treg cells that function to inhibit the proliferation and function of CD4^+^ T cells [24]–[26]. STAT5 activity among the CD4^+^ T cells in spleens was significantly increased by GSPE treatment (**Figure 2B**). Taken together, oral administration of GSPE in mice with obesity induced by high-fat diet resulted in significant suppression on Th17 cells and reciprocal induction of Treg cells. That results were associated with modulation of STAT proteins.

### Effect of GSPE in Obese-CIA mice

**Figure 3:**
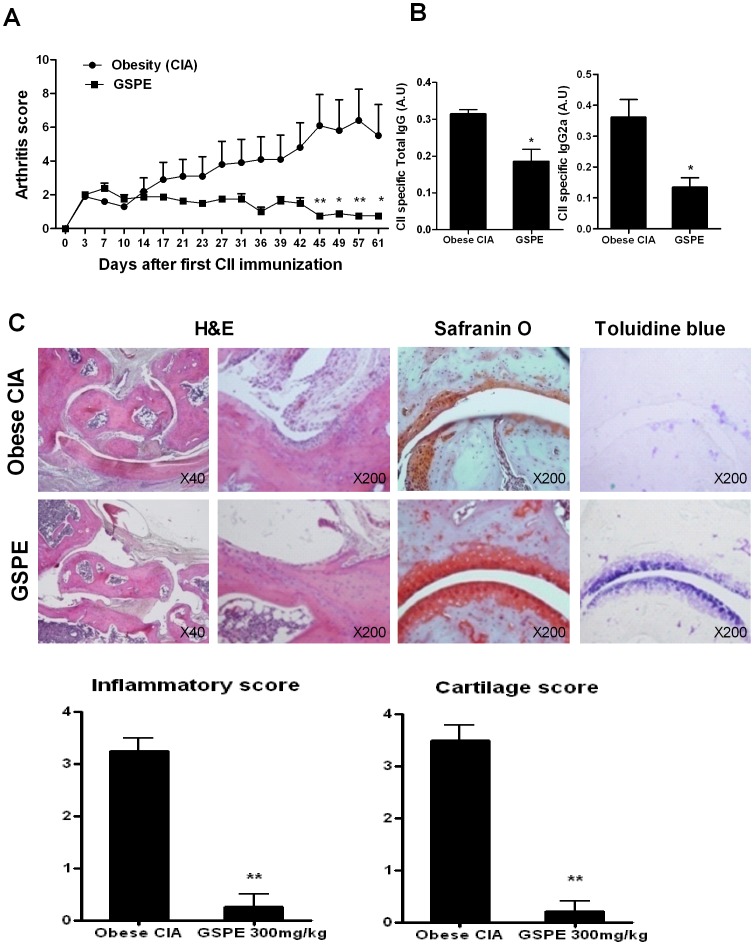
GSPE treatment attenuated arthritis severity in an obese CIA model (n = 6 for each group). First CII immunization in mice was conducted when the mouse weighed 30/kg GSPE dissolved in saline or saline only (control) every other day from day 14 after first CII immunization. (A) Arthritis score during the progression of obese CIA were evaluated. Error bars represented SEM. (B). Serum concentrations of CII specific total IgG and IgG2a were determined by ELISA in each group of mice. (C) Representative histopathologies of the ankle joints stained with H&E, Safranin O, and Toluidine blue in obese CIA mice. The joints from the mice treated with GSPE showed attenuated erosion and inflammation, whereas the joints from the mice treated with vehicle demonstrated markedly erosive and destructive arthritis (original magnification, 40×or 200×, as indicated). The mean histologic scores for inflammation and cartilage damage in mice treated with GSPE (n = 6 per group) or vehicle (n = 6) are shown in the graph. Error bars represented SD (*lower panel*). *P<0.05, **P<0.01 compared to the control-treated mice.

To induce obese CIA model in mice, C57BL/6 (wild-type) mice fed a high-fat diet (60 Kcal) were immunized with bovine CII. To ascertain the anti-arthritis effects of GSPE in obese CIA mice model, GSPE or control (saline) was given orally after CII immunization. The results showed that the mean arthritis score was significantly reduced in GSPE-treated arthritis group, compared with those of control-treated group. (**Figure 3A**). The serum concentrations of CII specific IgG and IgG2a were significantly decreased in GSPE-treated mice. The extents of joint inflammation and cartilage damage were assessed by H&E and safranin O and toluidine blue staining. In the obese-CIA mice, the joints showed the infiltration of inflammatory cells, synovial hyperplasia, and the destruction of the articular cartilage and bone. There was a statistically significant reduction in the inflammation scores of the GSPE-treated obese-CIA mice. The joints of obese CIA mice with GSPE treatment showed a significant reduction in cartilage loss, compared to the control group (**Figure 3C**).

### The anti-inflammatory effects of GSPE are associated with attenuated oxidative stress in the joints of obese CIA mice

**Figure 4:**
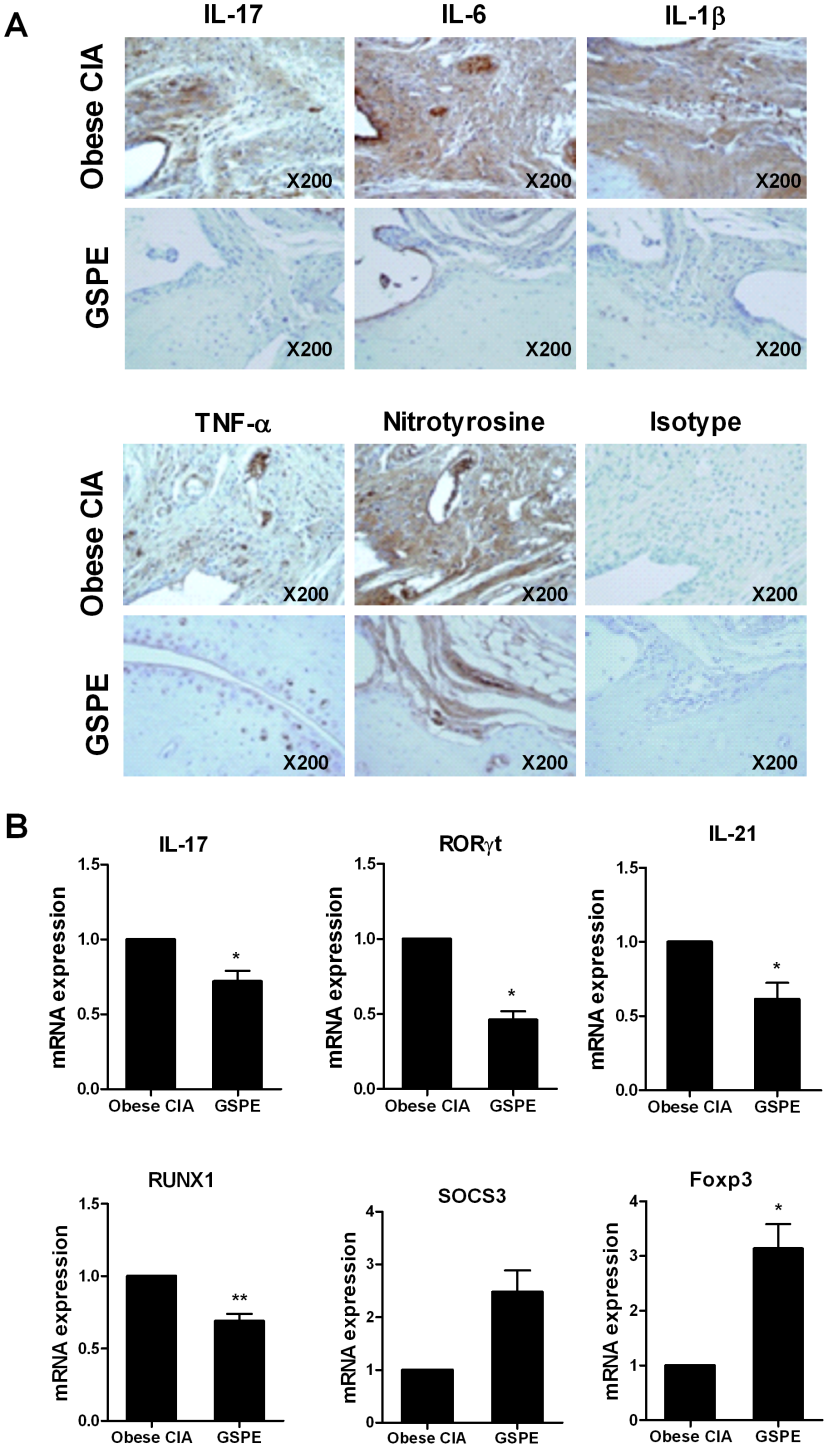
GSPE treatment decreases the expression of key pro-inflammatory molecules and modulates regulatory mediators in obese CIA mice (*n* = 6 for each group). (A) Representative histologic findings in joint sections from each group of mice. Joints sections were stained with anti-IL-17, anti-IL-6, anti-IL-1β, anti-TNF-α, anti-nitrotyrosine (an oxidative stress marker) antibodies and isotype (shown in brown). (B) The expressions of IL-17, RORγt, IL-21, RUNX1, SOCS3 and Foxp3 mRNA in splenocytes were determined by real-time PCR. *P<0.05, ** P<0.01 compared to the vehicle-treated group.

TNF-α, IL-1β, IL-6 and IL-17 are considered to be proinflammatory cytokines that are implicated in the pathogenesis of RA. We next investigated whether GSPE would affect the expression of the above molecules in the joints of obese CIA mice, by using immunohistochemistry. Compared with those of vehicle-treated group, the joints of GSPE-treated obese CIA mice demonstrated profoundly decreased cell population expressing TNF-α, IL-1β, IL-6 and IL-17 (**Figure 4A**). To determine the degree of oxidative damage to the joints, immunohistochemistry was used to assess the expression of nitrotyrosine on day 51 after CII immunization. The results showed that the expression of nitrotyrosine was significantly decreased in the joints of GSPE-treated obese CIA mice (**Figure 4A**). We examined the mRNA expressions of IL-17, RORγt, IL-21, RUNX1, SOCS3, and Foxp3 in the splenocyte isolated from both groups of mice. RUNX1 is the transcription factor that is involved in RORγt-dependent IL-17 production [27]. The mRNA expressions of IL-17, RORγt, IL-21 and RUNX1 were significantly decreased in the GSPE-treated group, whereas the expression of Foxp3 was increased. SOCS3 strongly suppresses STAT3 activity through IL-6 signaling [28]. The mRNA expression of SOCS3 tended to increase in GSPE-treated group, although the difference did not reach statistical significance (**Figure 4B**).

### GSPE–induced regulatory effect on Th17/Treg cell populations in obese CIA model is associated with activities of STAT3 proteins

**Figure 5:**
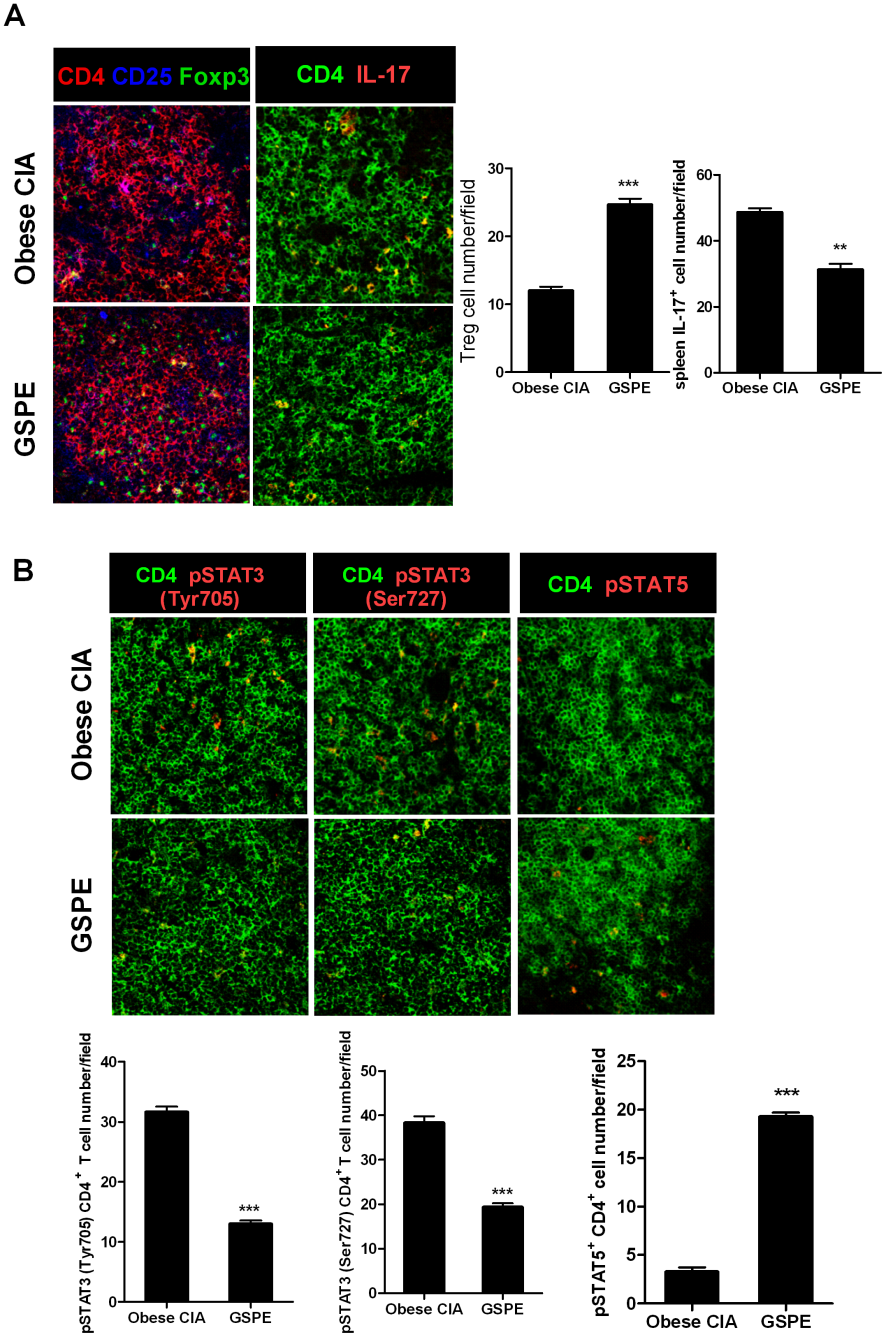
Treatment with GSPE decreased Th17 through *in vivo* regulation of pSTAT3^Tyr705^ and pSTAT3^Ser727^ and reciprocally increased Foxp3^+^ Treg cells through pSTAT5 induction in obese CIA mice (*n* = 6 for each group). (A) Spleen tissues from each group of mice were stained for CD4^+^CD25^+^Foxp3^+^ Treg cells and CD4^+^IL-17^+^ Th17 cells using monoclonal antibodies against CD4 (red), CD25 (blue), and Foxp3 (green) (*left image*) or CD4 (green) and IL-17 (red) (*right image*) (original magnification, 400×). CD4^+^CD25^+^Foxp3^+^ T cells and CD4^+^IL-17^+^ T cells were enumerated visually at higher magnification (projected on a screen) by four individuals, and the mean values are presented in the form of a histogram. **P<0.01, ***P<0.001 *versus* the vehicle-treated group. (B) Spleens from mice in each group were examined by immunofluorescence staining with monoclonal antibodies against CD4 (green) and pSTAT3^Tyr705^ (red) (*left image*), or CD4 (green) and pSTAT3^Ser727^ (red) (*middle image*), or CD4 (green) and pSTAT5 (*right image*) (original magnification, 400×). The CD4^+^pSTAT3^Tyr705^, CD4^+^pSTAT3^Ser727^ and CD4^+^pSTAT5^+^ cells were analyzed using laser confocal microscopy and the numbers of the cells were enumerated visually at higher magnification (projected on a screen) by four individuals. The mean values are presented in the form of a histogram. ***P<0.001 compared to the vehicle control mice.

To ascertain whether or not GSPE treatment in mice with obese CIA can control Th17/Treg cell population, each population of those T cell subset was assessed. The results showed a larger population of Foxp3^+^ Treg cells in GSPE-treated arthritis mice. In contrast with that, IL-17 expressing CD4^+^ T cells (mainly Th17 cells) were significantly decreased by treatment with GSPE (**Figure 5A**). In line with the results in DIO mice, GSPE treatment in arthritis mice also exhibited attenuated expressions of STAT3 activity (both pSTAT3^Tyr705^ and pSTAT3^Ser727^) in CD4^+^ T cells, whereas pSTAT5 activity in those cells was profoundly augmented (**Figure 5B**). We conclude that GSPE diverted differentiation of CD4^+^ T cells toward a Treg phenotype in murine model of obesity-associated autoimmune arthritis through a modulation of STAT3 proteins.

## Discussions

In this study, we showed that GSPE has a therapeutic effect on both obesity and obesity-associated arthritis. Oral administration of GSPE attenuated weight gain, lipid content in liver and serum glucose level, and improved lipid profiles in mice with obesity induced by high-fat diet. GSPE decreased the extent of oxidative stress in liver and spleens, assessed by nitrotyrosine expression. GSPE treatment in DIO mice accelerated the differentiation of Treg cells and reciprocally reduced that of Th17 cells *in vivo*. This deviation on T cell differentiation by GSPE treatment was identified by decreased STAT3 activity and increased STAT5 activity. Having the same mechanisms as obesity model, GSPE exhibited anti-inflammatory effects in obese CIA mice. Decreased population of Th17 cells through inhibition of STAT3 activity and increased Treg cells through induction of STAT5 activity were mainly implicated in immunomodulatory and anti-inflammatory effects of GSPE in obese CIA mice.

In line with our results, previous studies have suggested that GSPE could be an effective therapeutic agent for obesity. Pahuelo et al. showed that GSPE administration protected against weight gain in Wistar rats with obesity induced by cafeteria diet [5]. The action mechanism of GSPE in Wistar rats with obesity is regarded as improving the function of brown adipose tissue (BAT) mitochondria [5]. The activity of BAT has been revealed to be lower in overweight and obese individuals than in lean subjects [29], making BAT an interesting target for the treatment of obesity. Our study result presented that GSPE could treat obesity and metabolic disorders, of which mitochondria dysfunction is involved in the pathogenesis. In the present study, we had paid attention to other mechanisms of GSPE. When weight gain was reduced with GSPE, the capabilities of GSPE as an anti-inflammatory agent and a regulator of immune system were identified in our study.

We suggested the common signaling pathway and gene transcription between inflammation and obesity. Because STAT3 is a key transcription factor that is involved in Th17 differentiation, it is becoming a relevant treatment target for autoimmune diseases such as inflammatory bowel disease and RA [30], [31]. Interestingly, STAT3 is abundantly in adipocytes [32]. STAT3 signaling is also required for leptin regulation of energy [33]. The leptin receptor long form – STAT3 signal regulates food intake and energy expenditure by leptin. Furthermore, selective inhibitors of the JAK2-STAT3 signaling pathway suppressed adipogenesis *in vitro*, demonstrating a critical role of STAT3 activity in the modulation of adipogenesis [32]. Regarding STAT5, animal studies also have suggested that JAK2 and STAT5 proteins act to prevent hepatic lipid accumulation [34]–[36]. In the present study, GSPE promoted the significant reduction of STAT3 activity and the induction of STAT5 activity in spleens of the experimental obese mice, suggesting the inhibitory effects of GSPE on adipogenesis through modulation of STAT proteins.

Previously, we identified that GSPE could inhibits autoimmune arthritis via affecting T cell differentiation and cytokine secretion in CIA mice [18], [37]. In the previous studies, the anti-inflammatory property of GSPE was investigated on classical (lean) CIA mice. On the other hand, obese CIA mice model is a new animal model of autoimmune arthritis, which shows the amplified inflammatory responses, specifically through Th17 deviation. The number of Th17 cells and IL-17 mRNA expression of the splenocytes were higher in obese CIA mice than those of lean CIA animals [19]. The mechanism that obesity aggravated the inflammation of CIA mice was reversely the same as the immunological change after treatment with GSPE in obese CIA mice. Therefore, the anti-inflammatory effect of GSPE shown in this study is not an incidental or independent phenomenon, but an essential mechanism to treat exaggerated inflammation by obesity. This is the first study that demonstrated the therapeutic potential of GSPE in an experimental murine model of obesity (DIO) and obesity-associated autoimmune arthritis via induction of Treg differentiation and decreased differentiation of Th17 cells.

Obese CIA mice exhibited disturbed metabolic profiles; increased serum levels of fatty acid, glucose, and low density lipoprotein cholesterol compared to those of lean CIA animals (data not shown). However, the body weight of obese CIA mice tended to decrease compared to that of DIO mice. It could be proposed that accelerated inflammation in obese CIA mice caused weight loss in the animals. The inflammatory response in obesity has been considered to be a low-grade chronic inflammation, whereas RA has long been recognized as having a vigorous inflammatory component. Therefore, DIO mice and obese CIA mice may have some differences in the aspect of immunological phenotypes.

In conclusion, we demonstrated that GSPE could be an effective material on obesity and inflammatory diseases aggravated by obesity. GSPE acts to regulate T cell differentiation where it suppressed Th17 cells and reciprocally induced Treg cells in mice with obesity induced by high-fat diet and obese CIA mice. This deviation of T cells differentiation may be related with regulation of STAT proteins. Moreover, we established that GSPE is a promising substance for treating immunologic diseases related with STAT3 including several metabolic diseases, inflammatory diseases and neoplasms in the future.
